# Multifunctional nanoparticles in endodontics: applications, challenges, and future directions

**DOI:** 10.1186/s11671-025-04314-7

**Published:** 2025-08-07

**Authors:** Una Ivković, Catalina Moreno-Rabié, Arn Mignon, Mostafa EzEldeen, Reinhilde Jacobs

**Affiliations:** 1 OMFS-IMPATH Research Group, Department of Imaging and Pathology, Faculty of Medicine, University of Leuven, Leuven, Belgium; 2 Department of Oral and Maxillofacial Surgery, University Hospitals Leuven, Leuven, Belgium; 3 Smart Polymeric Biomaterials Research Group, Biomaterials and Tissue Engineering (SIEM), Campus Group T Leuven, University of Leuven, Leuven, Belgium; 4 Department of Oral Health Sciences, KU Leuven and Paediatric Dentistry and Special Dental Care, University Hospitals Leuven, Leuven, Belgium; 5 Department of Dental Medicine, Karolinska Institutet, Stockholm, Sweden

**Keywords:** Nanoparticles, Endodontics, Dental Materials, Biocompatible materials, Regeneration

## Abstract

Nanoparticles are emerging as transformative agents in endodontics, addressing challenges in treating the dentin-pulp complex. This scoping review aims to explore multifunctional applications of nanoparticles in endodontics, with a focus on their roles in promoting tissue regeneration through therapeutic effects, enhancing material properties, and serving a carrier function. Following PRISMA-Scoping Review guidelines, a comprehensive literature search was conducted across Web of Science, PubMed, and Scopus. A total of 490 articles were initially identified, of which 92 met the preliminary eligibility criteria. Following full-text screening, 70 studies were included in the qualitative synthesis. Key findings from both in vitro and in vivo studies are summarized in tabular form. Results reveal a notable imbalance in the types of nanomaterials studied: inorganic nanomaterials were reported in 77% of the studies, while only 23% investigated organic nanomaterials. Despite their lower representation, organic nanomaterials demonstrated considerable relevance. Chitosan was reported in 29% of the carrier studies, while extracellular vesicles were featured in 22% of the therapeutic applications. Among inorganic materials, bioactive glass was frequently reported, appearing in 31% of enhancer-related studies, 26% of therapeutic studies, and 13% of those investigating carrier functions. The end applications of these nanoparticles were in 69% of the studies either (direct) pulp capping or root canal filling, highlighting the need for innovative materials in these applications. Regarding experimental models, 75% of the studies conducted in vitro research on relevant cell lines, while 25% employed animal models. Of those 25% in vivo studies, 18% also reported in vitro findings. Nanoparticles hold significant promise for transforming endodontics, offering enhanced antibacterial efficacy and bioactivity while addressing critical limitations of conventional materials. However, challenges remain regarding their long-term biocompatibility, scalability, and integration into clinical workflows. This review emphasizes the need for translational research to bridge the gap between laboratory innovations and clinical practice.

**Graphical abstract:**

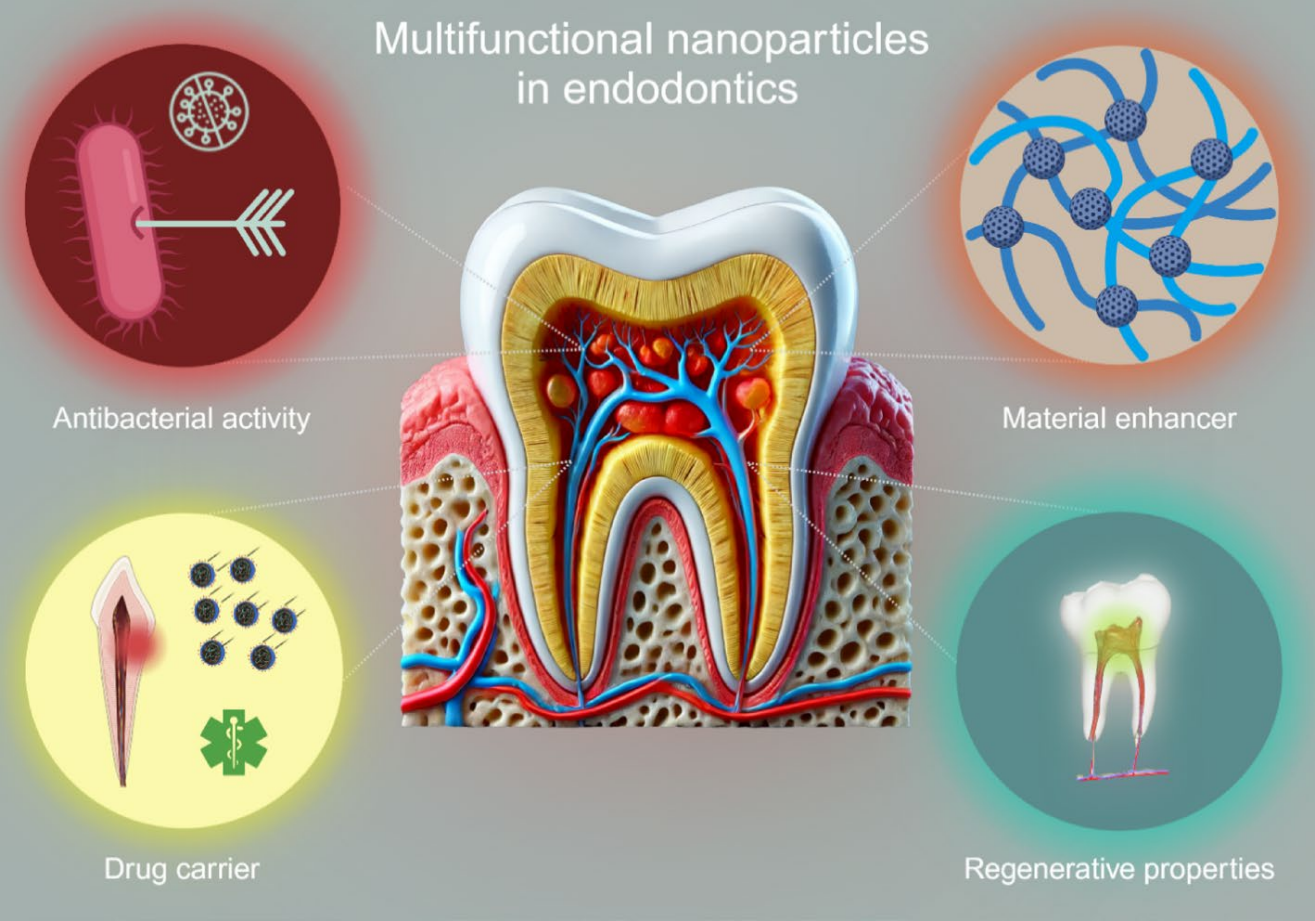

## Introduction

Biomaterials play a critical role in modern endodontics, serving as replacements for damaged tissues, delivery systems for bioactive molecules, and scaffolds for tissue regeneration [1, 2]. However, despite advances in materials science, the development of biomaterials that interact effectively with the intricate dentin-pulp complex remains a challenge. This complex microenvironment consists of multiple layers with different mechanical and physiological properties, requiring materials that balance durability, biocompatibility, and bioactivity [3]. Unfortunately, many conventional endodontic biomaterials fail to achieve this balance, limiting their clinical efficacy.

For example, while calcium silicate-based cements are widely used due to their bioactivity, they have drawbacks such as slow setting time, tooth discoloration, inconsistent mechanical properties, and suboptimal handling properties [4]. Similarly, current pulp capping agents and root canal sealers can promote tissue healing but lack sufficient antibacterial efficacy to prevent reinfection, a primary cause of endodontic treatment failure [5]. The limitations of these materials highlight the urgent need for innovative solutions that address not only mechanical and biological requirements, but also antimicrobial and regenerative potential.

Nanoparticles, defined as colloidal particles ranging between 1 and 100 nm, have already shown immense potential in several medical fields, including cancer treatment, wound healing, and endodontics [6–10]. They can be synthesised from a variety of materials and are often divided into two distinct groups: organic and inorganic nanoparticles [11]. In endodontics, nanoparticles could enhance the functional properties of biomaterials by improving mechanical strength, providing antibacterial effects to combat biofilm formation, and promoting stem cell differentiation [12, 13]. Their ability to act as carriers for therapeutic agents also enables innovative applications such as localised drug delivery and regenerative therapies. Antibacterial effects are critical in endodontics, where biofilm formation can compromise treatment outcomes [14]. These unique properties make nanoparticles valuable tools for addressing the complexities of the dentin-pulp complex and advancing endodontic biomaterials.

Even though the potential of nanoparticles has attracted considerable attention, existing reviews have mainly focused on their antibacterial properties [15, 16]. While studies have highlighted their efficacy in reducing biofilm-associated infections, their role in material reinforcement, regenerative endodontics, and drug delivery has been less systematically reviewed [17–20]. This gap highlights the need for a broader, more integrative perspective on nanoparticle applications in endodontics.

This scoping review aims to fill this gap by providing a comprehensive overview of the multiple roles of nanoparticles in the advancement of biomaterials in endodontics. It will explore their applications as material enhancers, carriers of therapeutic agents, and facilitators of tissue regeneration. The review synthesises findings from in vitro and in vivo studies, identifies limitations in current research, and outlines directions for future investigation. By addressing the challenges of the dentin-pulp complex, this scoping review aims to pave the way for the next generation of endodontic biomaterials and contribute to the wider adoption of nanoparticle-based therapies in clinical practice.

## Methods

### Protocol

Our protocol was drafted using the Preferred Reporting Items for Systematic Reviews and Meta-analysis Protocols (PRISMA) [21].

### Eligibility criteria

Published original research articles in English on applying nanoparticles in endodontics were considered eligible. Clinical studies were excluded due to the review’s focus on basic and preclinical research. Conference proceedings, recommendations, expert statements, technical reports, reviews, case reports, and non-original papers were excluded as well.

### Information sources

The databases used were Web of Science (WoS), PubMed, and Scopus. The primary literature search was performed on the 3rd of August 2023, and to ensure the inclusion of the most relevant and up-to-date literature, the databases were continuously monitored for newly published studies, and eligible articles meeting the inclusion criteria were manually added until the 13th of February 2025. This approach allowed for a comprehensive and current synthesis of the available evidence.

### Search

The search conducted was: ("Dental pulp" [Mesh] OR "Dental Pulp Cavity" [Mesh] OR "Dentin" [Mesh] OR "Endodontics" [Mesh] OR "Dental pulp exposure" [Mesh] OR "Dental Pulp Diseases" [Mesh] OR "Dental Pulp Capping" [Mesh] OR endodont* OR “pulp exposure” OR “pulp capping” OR “tooth” OR “dentine” OR “dental pulp” OR “dentistry”) AND ("Regenerative medicine" [Mesh] OR "Endodontic Regeneration" OR "Regenerative Endodontics" [Mesh] OR "Odontogenic differentiation" OR "Odontoblasts" [Mesh] OR "Cell Differentiation" [Mesh] OR “Pulp Regeneration” OR “dentinogenesis” OR “dentin formation”) AND ("Nanoparticles" [Mesh] OR "Nanoparticle Drug Delivery System" [Mesh] OR nanoparticle*) NOT (“Crowns” [Mesh] OR "Denture, Partial, Fixed" [Mesh] OR “Resin Cement” [Mesh] OR “Dental Cement” [Mesh] OR “Ceramics” [Mesh] OR “Dental Bonding” [Mesh] OR “Composite Resins” [Mesh] OR “post”). This search strategy was adapted to fulfill the requirements of different search engines.

### Selection of sources of evidence

After removing duplicates, both reviewers (U.I. and C.M.R.) screened the same publications independently. The articles were first screened on titles and abstracts. Following, a full-text screening was executed, where following exclusion criteria were used:

- Not referring to nanoparticles or unclear whether they do
- Applications other than cell differentiation, regeneration, or antimicrobial
- Studies related to bone
- No full text is available
- Poor quality
- Retracted article

Disagreements on study selection and data extraction were resolved by consensus and discussion.

### Data charting process

Two reviewers (U.I. and C.M.R.) jointly developed a data-charting form to determine which variables to extract. The two reviewers independently charted the data, discussed the results, and continuously updated the data-charting form in an iterative process.

### Data items

Data was abstracted on article characteristics (e.g., dental application), nanomaterial (e.g., bioactive glass, calcium phosphate, chitosan, silver, gold), used cell types, and results of the nanoparticle application (e.g., antibacterial activity, dental material enhancement or regeneration potential).

### Synthesis of the results

The studies were grouped by the function the nanoparticles fulfilled. They summarized the type of nanoparticles, cell types used, and experimental models for each paper, along with a concise effect of the nanoparticles. In the context of drug delivery, a carrier refers to nanoparticles designed to transport and release pharmaceutical compounds within the body. These nanosystems can target specific cells or tissues, ensuring that the drug is delivered precisely where needed, thereby increasing the efficacy of the treatment and reducing side effects. When functioning as an enhancer, nanoparticles are utilized to improve the properties of existing materials. By integrating nanoparticles, the material gains additional attributes such as increased strength, enhanced thermal stability, improved electrical conductivity, or better biocompatibility, which are beneficial for the intended application. As therapeutic agents, nanoparticles themselves exert a direct therapeutic effect on tissues. These nanoparticles can influence biological processes such as cellular differentiation, regeneration, or inflammation modulation, providing therapeutic benefits independent of any drug they might carry.

## Results

**Fig. 1:**
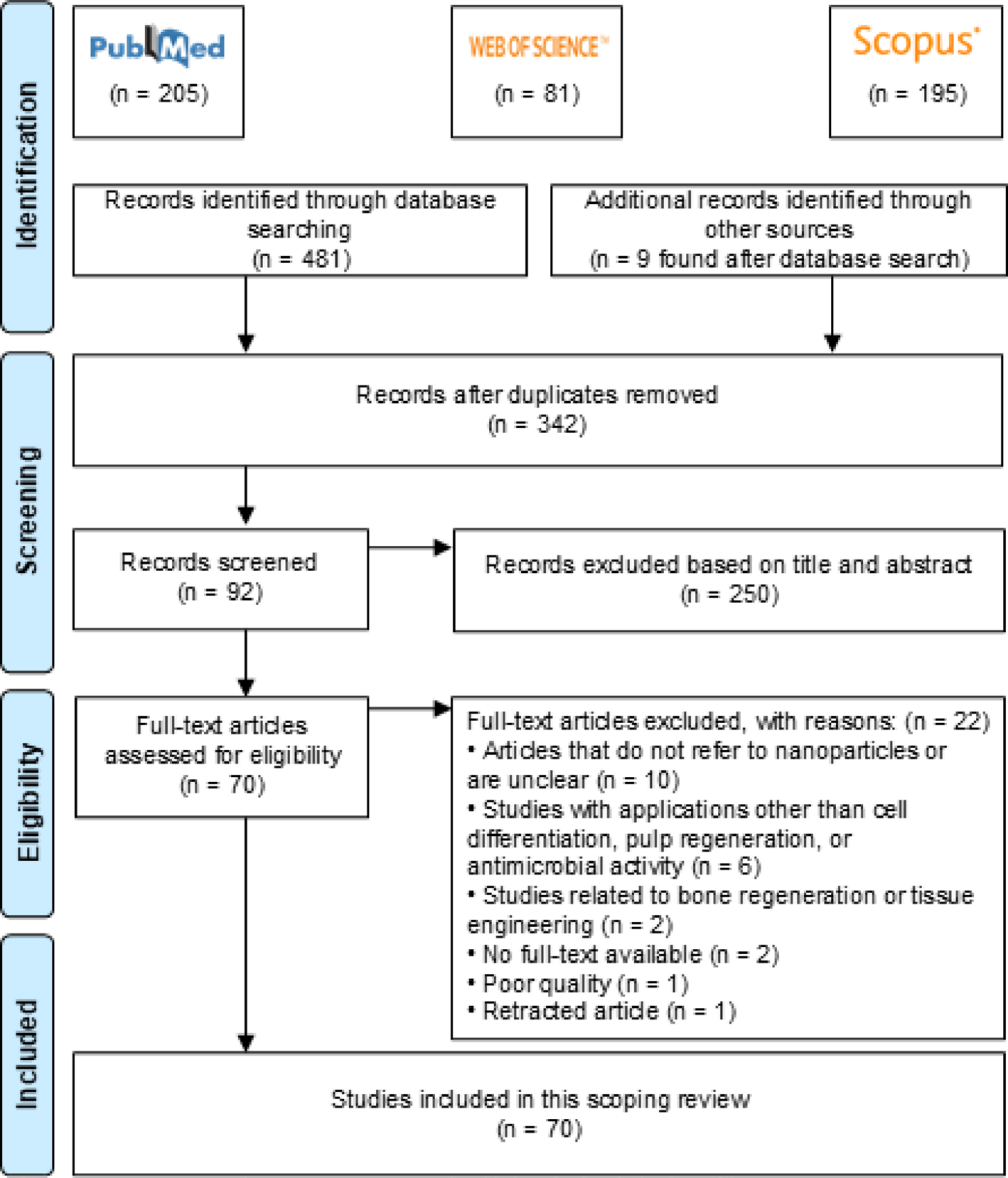
Overview of studies included in a PRISMA flow chart

A total of 490 papers were identified from database searches, with one additional paper identified manually. After removing duplicates, 342 papers were screened based on titles and abstracts. Of these, 92 full-text articles were assessed for eligibility, and 70 studies were ultimately included in the scoping review (Fig. 1). All included studies were published within the past two decades.

The studies were categorized based on the primary functions of the nanoparticles: carriers (n = 24), enhancers (n = 26), and therapeutic agents (n = 23). Notably, three studies reported dual functions, classifying nanoparticles as carriers and enhancers [22–24] or both carriers and therapeutic agents [24].

**Fig. 2:**
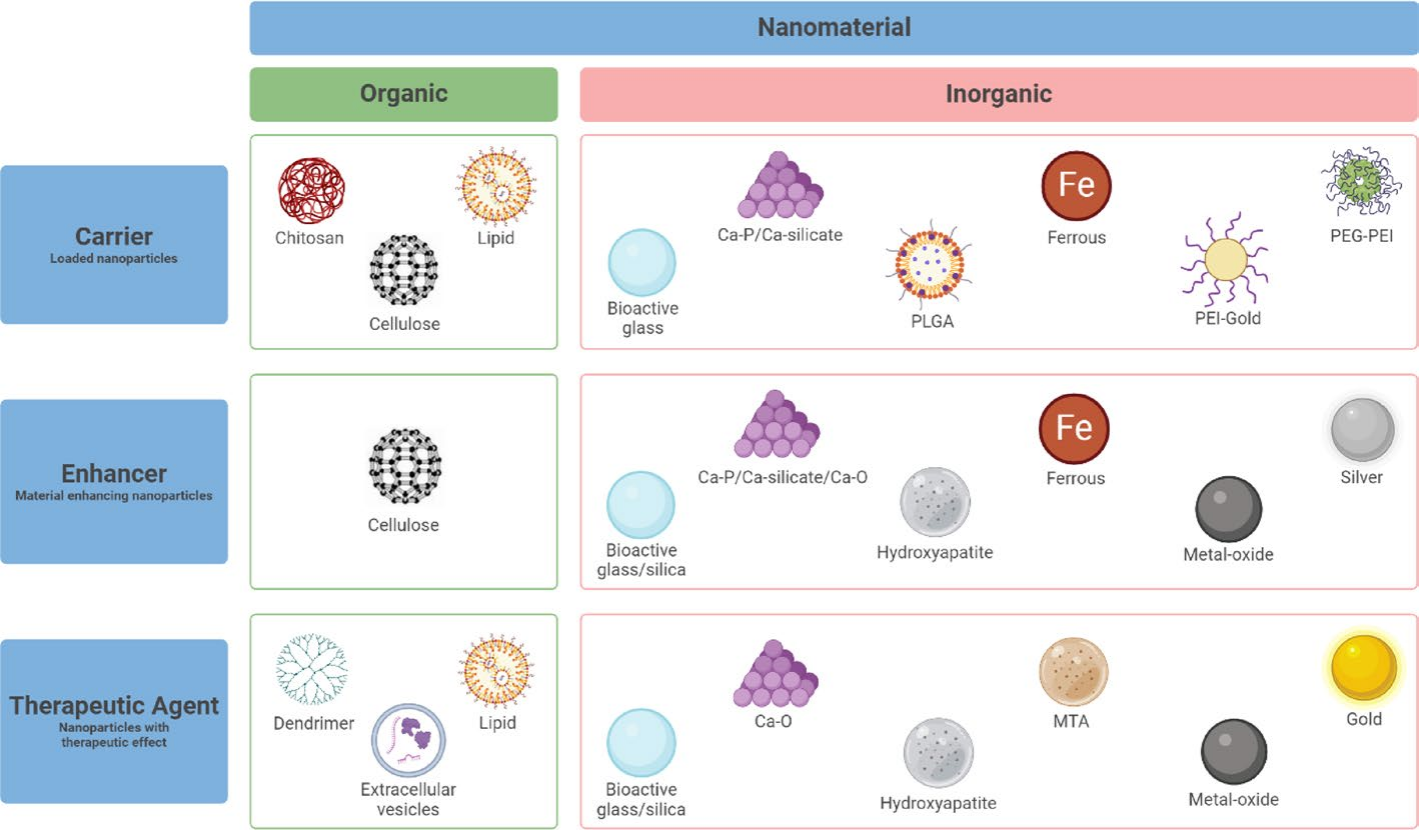
Overview of the primary functions of the nanoparticles and the reported nanomaterials

As shown in Fig. 2, the research landscape remains predominantly centred on inorganic nanomaterials (77%), while organic alternatives are investigated in 23% of the reported studies. Despite this disparity, organic nanomaterials demonstrate significant potential. Chitosan, for example, is featured in 29% of the studies focusing on drug delivery carriers, and extracellular vesicles are utilized in 22% of those targeting therapeutic applications. Among inorganic materials, bioactive glass stands out, appearing in 31% of enhancer-related studies, 26% of therapeutic studies, and 13% of those involving carrier functions.

The included studies reported a broad range of nanoparticle sizes, typically between 10 and 500 nm. However, Raddi et al. (2024) reported a substantially larger size range of 145 to 4200 nm, making it an outlier among the reviewed studies [25].

Out of the studies included, 75% reported in vitro experiments investigating various effects of nanoparticles on stem cells. These effects were validated based on experiments on differentiation, mineralisation capacities, cell viability, and regeneration occurrence. All studies reported odontogenesis to be the main dental application, except a few studies that had amelogenesis [26–28], antibacterial activity [29, 30], and angiogenesis [22] as the main dental application. Human dental pulp stem cells (hDPSCs) were the most frequently studied cell type, followed by stem cells of the apical papilla (SCAPs) [5, 31–35], stem cells from human exfoliated deciduous teeth (SHEDs) [36, 37], and periodontal ligament stem cells (PDLSCs) [38, 39]. Additional cell types included endometrial stem cells (EnSCs) [40], osteosarcoma stem cell lines such as SaOS2 cells [26] and MG63 [27, 41], bone marrow stem cells (BMSCs) [42], and periodontal ligament fibroblasts (PDLF) in co-culture with macrophages (Mφ) [43]. Four studies utilized rodent-derived stem cells, including rat DPSCs [44, 45], rat dental epithelial (HAT-7) cells [28], and RAW 264.7, an adherent cell line isolated from a mouse tumor [46].

Nanoparticles were also investigated for their antibacterial properties, with five studies focusing on their effects against common oral pathogens. These included *Escherichia coli (E. coli)* [41], *Enterococcus faecalis (E. faecalis)* [29, 47, 48], *Fusobacterium nucleatum (F. nucleatum)* [30], *Staphylococcus aureus (S. aureus)* [41], and *Streptococcus mutans (S. mutans)* [49].

Next to the in vitro studies, 25% demonstrated the effects of nanoparticles through in vivo investigations, and 18% of these studies combined in vitro and in vivo methodologies [23, 40, 43, 49–63]. Rat models were the most used, examining infected dental pulp tissue defects [50], pulp capping [54, 62], pulpotomy [58], pulpitis [63], diabetic pulp injury [61], and reimplantation [43]. Minipig models were used to study pulp injury [57] and capping [59]. Due to size restrictions to study the dental tissues, mouse models investigated subcutaneous ectopic implantation [51]. Other methods included ectopic implantation of human dentin in rat oral cavities [49], ectopic odontogenesis model and a tooth defect model in rats [23], and dorsum ectopic implantation [55, 60]. One study has reported an ex vivo design where the nanoparticle function was tested on the chick embryo chorioallantoic membrane (CAM) model [22].

The suggested end applications are summarised as well, where the future clinical application of the nanoparticles is elucidated (Table 1, column 7). Predominantly reported end applications are pulp capping materials, regeneration scaffolds, root canal fillings, and dentin conditioners. Fourteen studies had no unambiguous clinical application.

The results were summarized in three tables according to the primary function of the nanoparticles described. In Table 1A, nanoparticles that exhibit a carrier function were summarized. In Table 1B, nanoparticles enhancing existing dental materials were elaborated, while in Table 1C nanoparticle therapeutic properties were shown. These tables provide detailed insights into the materials, experimental models, and effects in vitro*/*in vivo reported across the studies, offering a comprehensive overview of nanoparticle applications in endodontics.

**Table 1:** Studies reporting nanoparticles in endodontics

References	Material	Average particle size (nm)	Application	Load	Dose (mg/ml)	Suggested end application	Experimental model	Effect
(A) Nanoparticles as carriers								
[64]	Bioactive glass	63	Odontogenesis	Dexamethasone	5–30	Scaffold (regeneration)	**hDPSCs**	Odontogenesis ↑
[50]	Bioactive glass	60	Odontogenesis	Tetracycline	1.25–10	Antimicrobial scaffold	**hDPSCs**	Biofilm formation ↓
			Antibacterial	Chlorhexidine digluconate	25		*Rat*	Odontogenesis ↑
[49]	Bioactive glass	400	Antibacterial	Epigallocatechin-3-gallate	2	Protection of exposed dentin	***S. mutans***	Biofilm formation ↓
			Odontogenesis				**hDPSCs**	Odontogenesis ↑
							*Rat*	Biomineralisation ↑
[23]	Bioactive glass	–	Odontogenesis	Simvastatin	0.25–4	Pulp capping	**hDPSCs**	High loading capacity ✓
	*See also Table B						*Rat*	
[22]	CNC	57–121	Angiogenesis	Platelet-derived GF	0.001–0.008	Regeneration hydrogel	**hDPSCs**	Sustained release ✓
	*See also Table B			Vascular endothelial GF			Fertilized chicken eggs	Neovascularization ↑
[51]	Calcium phosphate	75–100	Odontogenesis	*BMP-2*	–	Pulp capping	**hDPSCs**	Cell viability ↑
				*DMP-1*				No adverse effects ✓
							*Mouse*	Odontogenesis ↑
[44]	Calcium phosphate	200	Odontogenesis	*Bmp2*	–	–	**rDPSCs**	Non-viral vector ✓
								Odontogenesis ↑
[47]	Calcium silicate	130–154	Antibacterial	Gentamicin	1.1	Root canal filling	***E. faecalis***	Antibacterial activity ✓
			Odontogenesis	Fibroblast GF-2			**hDPSCs**	Odontogenic-related protein ↑
[31]	Chitosan	112–180	Odontogenesis	BSA	1.5	–	**SCAPs**	Temporal-controlled release ✓
								Dentin-pulp regeneration ↑
[5]	Chitosan	59	Odontogenesis	Dexamethasone	0.005	–	**SCAPs**	Sustained release ✓
								Odontogenesis ↑
[32]	Chitosan	112–179	Odontogenesis	TGF-β1	1.5	Regeneration scaffold	**SCAPs**	Release ✓
								Migration + differentiation SCAPs ↑
[33]	Chitosan	112–179	Odontogenesis	Dexamethasone	1.5	Dentin conditioner	**SCAPs**	Sustained release ✓
								Odontogenesis ↑
[34]	Chitosan	197–262	Odontogenesis	Dexamethasone	–	Dentin conditioner	**SCAPs**	Sustained release ✓
								Cell viability, migration, differentiation ↑
								Inflammation ↓
[43]	Chitosan	–	Odontogenesis	Dexamethasone	0.03	Root modification agents	**PDLF, Mφ**	Clastic differentiation ↓
				Rose Bengal	0.1		*Rat*	Resorption and ankylosis ↓
[65]	Chitosan-polylactic acid	100–300	Odontogenesis	Fluocinolone acetonide/coumarin-6	5.8	Pulp capping	**DPSCs**	Inflammation ↓
				BSA/BMP-2	8			Odontogenesis ↑
[28]	Lipid	–	Amelogenesis	*Tbx1*	0.2	–	**rHAT-7**	Amelogenesis ↑
								Enamel-like tissue formation ↑
								Efficient delivery method
[25]	Lipid	145–4200	Antibacterial	Chlorhexidine	4–10% w/v	Pulp capping	**L929 Murine Fibroblasts**	Sustained release ✓
								Cell Viability ✓
								Antibacterial activity ✓
[66]	Methacrylic acid	–	Osteogenesis	Tideglusib	0.0017	Pulp capping	**DPSCs**	Osteogenensis ↑
								Mineralisation ↑
[67]	Ferrous material	–	Dentinogensis	miR-218	–	–	**DPSCs**	miR-218 ↓ dentinogenesis of DPSCs
								miR-218 ❌ → mineralisation ↑
[62]	Cerium oxide	30	Odontogenesis	DMP-1	0.001	Pulp capping	**DPSCs**	Reparative dentin formation ✓
							*Rat*	
[68]	PEG-PEI	150–360	Odontogenesis	miR146a/bFGF	–	Vital pulp therapy	**hDPSCs**	Sustained release ✓
								Cell proliferation ↑
								Odontogenesis ↑
[52]	PEI-Gold	30	Odontogenesis	AntagomiR-3074-3p	–	Pulp capping	**hDPSCs**	Restorative dentin ↑
							*Rat*	Odontogenesis ↑
[53]	PLGA	–	Odontogenesis	Lovastatin	0.1	Direct pulp capping	**hDPSCs**	Cytotoxicity lovastatin ↓
							*Rat*	Tubular reparative dentin ↑
[24]	EV	30–150	Odontogenesis	RUNX3	–	–	**DPSCs**	Odontogenic differentiation ↑
	*See also Table C							

Reference	Material	Average particle size (nm)	Application	Enhanced material	Suggested end application	Experimental model	Effect
(B) Nanoparticles as enhancers							
[23]	Bioactive glass	–	Odontogenesis	Cement	Regeneration scaffold	**hDPSCs**	Biocompatibility ↑
	*See also Table A					*Rat*	Odontogenic potential ↑
[69]	Bioactive glass	–	Odontogenesis	PCL/gelatine composite	Scaffold	**hDPSCs**	Odontogenesis ↑
[70]	Bioactive glass	80	Odontogenesis	Calcium phosphate cements	Pulp capping	**hDPSCs**	Odontogenesis ↑
							Angiogenesis ↑
[71]	Bioactive glass	–	Odontogenesis	Cellulose acetate/oxidized pullulan/gelatine-based scaffolds	Pulp capping	**hDPSCs**	Cell attachment ↑
							Odontogenesis ↑
[72]	Bioactive glass	300–600	Odontogenesis	Graphene oxide composites	Composite filling	**hDPSCs**	Odontogenesis ↑
[73]	Bioactive glass	–	Odontogenesis	Biodentine (Calcium silicate cement)	Root canal preparation	**hDPSCs**	Attachment and proliferation ↑
							ALP expression ↑
							Mineralisation ↑
[41]	Bioactive glass	148–172	Antibacterial	Composite or cement	–	***S. aureus***	MBGN antimicrobial activity ↑
	Calcium silicate	Agglomerates	Odontogenesis			***E. coli***	TCS bioactivity ↑
						**MG63**	
[74]	Bioactive glass	–	Odontogenesis	Calcium phosphate cement	Pulp capping	**hDPSCs**	Cell proliferation & adhesion ↑
			Angiogenesis				Odontogenesis ↑
							ALP activity & expression odontogenic genes ↑
[75]	Calcium phosphate	20–30	Odontogenesis	Resin-modified glass ionomer cements	Pulp capping	**hMSCs**	Biocompatibility ↑
							ALP activity ↑
							Odontogenesis ↑
[54]	Calcium phosphate	116 (refers to previous study [76])	Antimicrobial	Composite	–	*Rat*	Pulpal inflammation ↓
			Odontogenesis	Adhesive			Tertiary dentin formation ↑
[63]	Calcium phosphate-Zinc phosphate	200	Anti-inflammation	Sodium alginate	Pulp capping	**DPSCs**	Inflammation resolution↑
			Odontogenesis			*Rat*	Dentin mineralization ↑
[36]	Calcium silicate	100	Odontogenesis	Biodentine (calcium silicate cement)	Pulp capping	**SHEDs**	Odontogenesis ↑
[77]	Calcium silicate	–	Odontogenesis	iRoot FS (calcium silicate cement)	Root canal filling	**hSCAPs**	Cell migration ↑
							Osteo/odontogenesis ↑
[22]	CNC	57–121	Angiogenesis	Hyaluronic acid	Regeneration hydrogel	**hDPSCs**	Material stability ↑
	*see also Table A					Fertilized chicken eggs	
[78]	Hydroxyapatite	W 17–23	Odontogenesis	PCL nanofibrous composite scaffold	Pulp capping	**DPSCs**	Cell viability and adhesion ↑
		H 93–146					Odontogenesis ↑
[79]	Ferrous material	10	Odontogenesis	Magnetic nanofiber scaffold	Regeneration scaffold	**hDPSCs**	Cell growth ↑
			Angiogenesis				Odontogenic differentiation ↑
							Pro-angiogenesis ↑
[80]	Iron oxide	38–246	Odontogenesis	GelMA/PEGDA composite hydrogel	Regeneration hydrogel	**DPSCs**	Osteo/odontogenic differentiation ↑
[81]	Ferrous material	–	Odontogenesis	Magnetic nanofiber scaffold	Regeneration scaffold	**hDPSCs**	Cell migration ↑
							Odontogenesis ↑
[82]	Calcium oxide	200–340	Odontogenesis	SAPO-34 zeolite and chitosan composite	Regeneration scaffold	**hDPSCs**	Cell proliferation ↑
	Iron oxide						Osteogenic differentiation ↑
[83]	Silica	30–60	Odontogenesis	Calcium silicate cement	Pulp capping	**hDPSCs**	Cell viability ↑
							Odontogenic marker level ↑
[30]	Silver	10	Antibacterial	Calcium hydroxide	Irrigant/ intracanal treatment	***F. nucleatum***	Combined effect is more efficient
[38]	Silver	–	Odontogenesis	Calcined tooth powder	Pulp capping	**Canine PDLSCs**	Odontogenic & neuronal differentiation ↑
[29]	Silver	25	Antibacterial	Methylcellulose gel	Intracanal medicament	***E. faecalis***	Biofilm reduced and eliminated
[48]	Silver	–	Antibacterial	Methacrylated gelatine	Pulp capping	***E. faecalis***	Antibacterial activity ✓
			Angiogenesis			**DPSCs**	Angiogenesis ↑
[40]	Titanium oxide	< 50	Odontogenesis	Chitosan	Direct pulp capping	**EnSCs**	Dentin repair ↑
						*Rat*	
[84]	Zinc-oxide	–	Odontogenesis	Gutta-Percha	Root canal filling	**hDPSCs**	Hydroxyapatite deposition ↑

Reference	Material	Average particle size (nm)	Application	Suggested end application	Experimental model	Effect
(C) Nanoparticles as therapeutic agents						
[85]	Bioactive glass	–	Odontogenesis	Protection of exposed dentin	**hDPSCs**	Odontogenesis ↑
[55]	Bioactive glass	20	Odontogenesis	Pulp capping	**hDPSCs**	Odontogenesis ↑
					*Mouse*	Dentin formation ↑
[86]	Bioactive glass	24–44	Odontogenesis	–	**hDPSCs**	Odontogenesis ↑
[37]	Bioactive glass	160	Odontogenesis	–	**SHEDs**	Odontogenic & dentin regeneration ↑
[45]	Bioactive glass	300–500	Odontogenesis	Pulp capping	**rDPSCs**	Odontogenesis ↑
[26]	Bio-silica	30	Amelogenesis	–	**SaOS-2 cells**	Hydroxyapatite deposition ↑
						Biomineralisation ↑
[87]	Calcium phosphate lipid	100–500	Odontogenesis	Pulp capping	**hDPSCs**	Inflammation ↓
						Odontogenesis ↑
[56]	Calcium oxide	15–65	Odontogenesis	Tooth & pulp-dentin complex development	*Rat*	Predentin & periodontal ligament thickness ↑
						Vascularization in pulp tissue ↓
[46]	Calcium oxide	–	Odontogenesis	Intracanal medicament	**RAW 264.7**	Osteoclast
	Calcium oxide loaded PLGA					differentiation ↓
[35]	Copper-oxide	–	Odontogenesis	Regenerative endodontics	**SCAPs**	Osteogenic/Odontogenic differentiation ↑
[57]	EV	50–150	Odontogenesis	Pulp capping	*Minipig*	Odontoblast related protein ↑
						Formation continuous reparative dentin ↑
[88]	EV	80–200	Odontogenesis	Regeneration hydrogel	**DPSCs**	Angiogenesis ↑
[39]	EV	< 200	Odontogenesis	–	**PDLSCs**	Osteo/odontogenic differentiation ↑
[60]	EV	100–150	Odontogenesis	Regeneration hydrogel	**DPSCs**	Odontoblastic differentiation ↑
					*Rat*	
[24]	EV	30–150	Odontogenesis	–	**DPSCs**	Odontogenic differentiation ↑
	*See also Table A					
[42]	Gold	34	Odontogenesis	Dental tissue engineering and odontoblastic differentiation	**DPSCs**	Endogenous stem cells ↑
					**BMSCs**	Dental pulp regeneration ↑
[58]	Hydroxyapatite	–	Odontogenesis	Direct pulp capping	*Rat*	Formation dentinal bridge containing dentinal tubules ↑
[59]	Hydroxyapatite	–	Odontogenesis	Direct pulp capping/ Pulpotomy	*Pig*	Inflammatory reaction ❌
[27]	Hydroxyapatite	–	Amelogenesis	–	**MG63**	Enamel remineralisation ↑
	Hydroxyapatite	W 17–23	Odontogenesis	Pulp capping	**DPSCs**	Immunomodulatory genes ↑
		H 93–146				
[89]	MTA powder	41	Odontogenesis	Pulp capping	**DPSCs**	Odontogenesis ↑
[90]	PAMAM	–	Odontogenesis	–	**DPCs**	Odontogenesis ↑
[61]	Cerium oxide (hyaluronic acid modified)	182.8	–	Pulp capping	**DPSCs**	Diabetes-induced dental pulp damage ↓
					*Mouse*	
[91]	Titanium dioxide	29	Odontogenesis	–	**DPSCs**	Biomineralisation ↑

CNC: cellulose nanocrystals; GF: Growth Factor; BMP: Bone Morphogenetic Protein; DMP: dentin matrix protein; BSA: bovine serum albumin; TGF: Transforming growth factor; PDLF: periodontal ligament fibroblasts; Mφ: macrophages; *Tbx*: T-box gene; PEG-PEI: polyethylene glycol-polyethylene imine; PEI, polyethylene imine; PLGA: poly(lactic-co-glycolic acid); PCL: polycaprolactone; GelMA: Methacrylated gelatine, PEGDA: Poly(ethylene glycol) diacrylate, MTA: mineral trioxide aggregate; W: width; H: height; nHA(EA): nanohydroxyapatite (from *Elaeagnus angustifolia*); EV: extracellular vesicles. Experimental model: Bold, in vitro; Italic, in vivo; Underline, ex vivo

## Discussion

This review comprehensively explored the versatile roles of nanoparticles in endodontics, emphasizing their potential to enhance cell differentiation, improve antimicrobial activity, and address the challenges posed by the complex dentin-pulp structure. Nanoparticles, classified as drug carriers, material enhancers, or therapeutic agents, offer significant promise in advancing endodontic treatments and paving the way for innovative regenerative approaches.

Nanoparticles provide a highly effective platform for delivering therapeutic agents by encapsulating, adsorbing, or dissolving drugs within their matrix [6]. These therapeutic agents include antimicrobials such as gentamicin [47] and tetracycline [50], while growth factors like fibroblast growth factor 2 (FGF-2) [47], platelet-derived growth factor (PDGF), and vascular endothelial growth factor (VEGF) [22], have shown potential to promote angiogenesis and tissue regeneration. Moreover, the gene *Tbx1* [28] is essential for ameloblast differentiation, ensuring normal tooth development, while gene *BMP-2* was encapsulated for its ability to stimulate the differentiation of hDPSCs into odontoblasts [44, 51]. Polymeric nanoparticles have prominently been reported in the function of a carrier in dental applications. Chitosan nanoparticles have gained prominence thanks to their cationic nature, which facilitates spontaneous assembly and protects sensitive agents from degradation [92]. Beyond endodontics, chitosan nanoparticles have shown potential in other fields, such as anti-diabetes therapy [93], Crohn’s disease management [94], and oncological treatment [95]. These findings highlight their versatility and promise in clinical applications.

Enhancing the properties of dental materials represents another critical application of nanoparticles. Bioactive glass, widely studied for its ionic dissolution of phosphorus, calcium, and silicate ions [96] and its ability to promote hydroxyapatite formation, has proven effective in integrating hard and soft tissues [69, 97]. Bioactive glass also has antibacterial effects when doped in metal ions such as silver, making it a popular choice for endodontic materials [98]. Additionally, green-synthesised nanohydroxyapatite, created using plant extracts, has demonstrated the potential to control nanoparticle morphology while promoting angiogenesis and odontogenesis [78, 99]. These plant-derived hydroxyapatite nanoparticles offer antioxidant and anti-inflammatory properties, further enhancing their therapeutic benefits and making them a promising option for sustainable biomaterial development. Moreover, they stimulate odontogenic differentiation of DPSCs, as an upregulation of odontogenic and osteogenic gene expression was measured. The presence of phenolic compounds and flavonoids in the plant extracts can raise Ca^2+^ deposition as well as activate osteoblast differentiation [100, 101].

Incorporating antibacterial properties into endodontic materials is critical for preventing infections and ensuring successful treatment outcomes. Silver nanoparticles have been extensively studied for their bactericidal properties, primarily attributed to the release of Ag + ions [8]. However, their clinical application remains limited due to biocompatibility issues and the risk of tooth discoloration [9]. Bioactive glass also demonstrates antibacterial potential, though its mechanisms require further elucidation [102]. Other papers reported that the antibacterial effects come from the increased dissolution tendency, leading to higher pH and alkali ion concentrations, which is harmful to the bacteria [102]. Strengthening the antibacterial properties of nanoparticles while addressing these limitations remains a vital area for future research.

Beyond their role in enhancing materials, nanoparticles themselves act as therapeutic agents. Bioactive glass, known for its mineralisation-inducing capabilities, is a leading example [103]. Emerging evidence also points to the therapeutic potential of extracellular vesicles (EVs), which are secreted by mesenchymal stem cells and carry bioactive molecules such as growth factors and chemokines [104]. EVs have demonstrated pro-angiogenic effects and the ability to induce odontogenesis and bone regeneration when derived from specific stem cells [105]. Their potential for patient-specific applications positions them as promising agents for personalized regenerative therapies, though ethical and economic challenges must be addressed.

Despite their promise, several critical issues in nanoparticle research require attention. Many studies focus on enhancing the differentiation of human dental pulp stromal/stem cells (DPSCs), given their role in maintaining and repairing the dentin-pulp complex. DPSCs, which have neurovascular and multi-differentiation properties, are a vital target for regenerative approaches, making them a primary focus of nanoparticle research [106]. However, the heterogeneity of this cell population and the complexity of this tissue remains an understudied topic that needs to be considered before the translation of research results [107]. Moreover, for nanoparticles functioning as drug carriers, precise dosage and particle size reporting is essential to ensure therapeutic efficacy and reproducibility [108, 109]. Notably, seven out of 24 studies categorized under drug carriers (Table 1A) did not report dosage, undermining the reliability of their findings [24, 34, 44, 51, 52, 67, 68]. Similarly, accurate classification of nanoparticle morphology is crucial, as non-spherical structures such as nanorods have occasionally been misclassified, leading to potential misinterpretation [78, 99].

The choice of stem cells is critical for ensuring relevance in regenerative studies. Dental-origin stem cells, such as DPSCs, PDLSCs, and SHEDs, are more directly applicable to endodontic research than non-dental stem cells like endometrial [40] or osteosarcoma stem cells [26, 27, 41]. Researchers must provide strong justification for using non-dental stem cells to avoid introducing irrelevant variables and ensure findings are translatable to dental applications. Similarly, the reliance on traditional in vitro models, while useful, often fails to replicate the complexity of the oral environment. Advanced 3D in vitro and ex vivo models, such as whole tooth models, offer more physiologically relevant alternatives [110]. Additionally, in vivo studies using animal models, such as rats or minipigs, are essential for validating findings before transitioning to clinical trials.

The antibacterial properties of nanoparticles remain a focal point in endodontics, yet most studies focus on single bacterial strains, limiting the generalizability of their findings [50]. Expanding antibacterial testing to include multispecies biofilms and microbiome-based approaches would better reflect the complexities of the oral environment and improve the clinical relevance of the results.

Finally, while nanoparticles have been explored in clinical trials for periodontal and orthodontic applications, their use in endodontics remains limited [111–114]. Only two randomized clinical trials have explored the antibacterial properties of nanoparticles in endodontics, demonstrating promising outcomes in bacterial reduction and pain relief [115, 116]. However, further research is needed to expand nanoparticle applications to other therapeutic areas, such as tissue regeneration and personalized endodontic treatments. Future studies must address scalability, long-term safety, and cost-effectiveness challenges to realize the full potential of nanoparticles in clinical practice.

This review highlights nanoparticles' transformative potential in addressing endodontics' challenges, particularly in regenerative approaches. However, bridging the gap between laboratory innovation and clinical application requires sustained efforts to standardize methodologies, optimize therapeutic strategies, and evaluate long-term outcomes in diverse patient populations.

## Conclusion and future perspectives

Traditional endodontic treatments often rely on the removal of pulp tissue and its replacement with bio-inert materials, a practice that, while effective in the short term, may lead to long-term complications such as structural weakness and reinfection. The need for biologically compatible, tissue-preserving solutions is becoming increasingly urgent, particularly as the field shifts toward regenerative approaches. A multi-modal strategy that integrates biomaterials capable of mimicking the native properties of the dentin-pulp complex offers significant promise in overcoming these limitations.

Nanoparticles have emerged as pivotal tools in advancing this strategy, offering unique advantages in enhancing the mechanical and biological properties of endodontic materials. Their role as carriers for therapeutic agents, including antimicrobials and regenerative factors, enables targeted and localized treatments, potentially transforming the standard of care. Additionally, their ability to enhance material performance and promote tissue repair makes them invaluable in addressing the challenges of regenerating the complex dentin-pulp structure.

Despite the substantial progress demonstrated by the studies reviewed, significant challenges remain. Many findings are derived from controlled laboratory settings, which often fail to replicate the complexity of the oral environment. Advanced in vitro systems, such as 3D models, and in vivo studies using animal models are essential to validate these promising results. Additionally, microfluidic tissue-on-chips and organ-on-chips models are becoming increasingly important in validating results and providing more physiologically relevant data [117]. Moreover, scaling these findings for clinical application requires addressing issues such as long-term safety, biocompatibility, and cost-effectiveness.

Future research should prioritize bridging the gap between laboratory innovation and clinical application. Key areas of focus include optimizing nanoparticle formulations for specific therapeutic goals, improving the reproducibility of findings through standardized methodologies, and expanding antibacterial testing to reflect the multispecies nature of the oral microbiome. Additionally, clinical trials are needed to evaluate the real-world efficacy of nanoparticle-enhanced materials and their potential to improve patient outcomes.

Incorporating nanoparticles into endodontic practice represents a transformative opportunity to advance the field from bio-inert to bioactive treatments. By fostering tissue preservation, promoting regeneration, and enabling targeted therapies, nanoparticles offer a pathway to revolutionize endodontic care and significantly improve long-term outcomes for patients.

## Data Availability

No datasets were generated or analysed during the current study.
